# Assessment of the Growth and Reproductive Performance of Cloned Pietrain Boars

**DOI:** 10.3390/ani10112053

**Published:** 2020-11-06

**Authors:** Junsong Shi, Baohua Tan, Lvhua Luo, Zicong Li, Linjun Hong, Jie Yang, Gengyuan Cai, Enqin Zheng, Zhenfang Wu, Ting Gu

**Affiliations:** 1 National Engineering Research Center for Breeding Swine Industry, College of Animal Science, South China Agricultural University, Guangzhou 510642, China; junsongstone@wens.com.cn (J.S.); tanbaohua@stu.scau.edu.cn (B.T.); lizicong@scau.edu.cn (Z.L.); linjun.hong@scau.edu.cn (L.H.); jieyang@scau.edu.cn (J.Y.); cgy0415@scau.edu.cn (G.C.); eqzheng@scau.edu.cn (E.Z.); 2 Guangdong Wens Breeding Swine Technology Co., Ltd., Yunfu 527300, China; lvye345@163.com

**Keywords:** SCNT, pig, reproductive performance, growth performance

## Abstract

**Simple Summary:**

Somatic cell nuclear transfer (SCNT) is a potential and promising technique for preserving and enlarging superior livestock genetic resources. Here, we investigated the effects of cloning on Pietrain boars, which are widely used to produce lean-type swine because of their high growth rate and lean fat rate. Our data revealed that SCNT manipulation caused some defects in growth performance, but the clones had normal semen quality and reproductive performance. Furthermore, the progeny of clones exhibited better growth performance than non-clones. These results indicated that the cloning of superior boars can be applied to produce more commercial pigs with increased economic benefit in practical breeding and production.

**Abstract:**

How to maximize the use of the genetic merits of the high-ranking boars (also called superior ones) is a considerable question in the pig breeding industry, considering the money and time spent on selection. Somatic cell nuclear transfer (SCNT) is one of the potential ways to answer the question, which can be applied to produce clones with genetic resources of superior boar for the production of commercial pigs. For practical application, it is essential to investigate whether the clones and their progeny keep behaving better than the “normal boars”, considering that in vitro culture and transfer manipulation would cause a series of harmful effects to the development of clones. In this study, 59,061 cloned embryos were transferred into 250 recipient sows to produce the clones of superior Pietrain boars. The growth performance of 12 clones and 36 non-clones and the semen quality of 19 clones and 28 non-clones were compared. The reproductive performance of 21 clones and 25 non-clones were also tested. Furthermore, we made a comparison in the growth performance between 466 progeny of the clones and 822 progeny of the non-clones. Our results showed that no significant difference in semen quality and reproductive performance was observed between the clones and the non-clones, although the clones grew slower and exhibited smaller body size than the non-clones. The F1 progeny of the clones showed a greater growth rate than the non-clones. Our results demonstrated through the large animal population showed that SCNT manipulation resulted in a low growth rate and small body size, but the clones could normally produce F1 progeny with excellent growth traits to bring more economic benefits. Therefore, SCNT could be effective in enlarging the merit genetics of the superior boars and increasing the economic benefits in pig reproduction and breeding.

## 1. Introduction

Somatic cell nuclear transfer (SCNT, also known as cloning) is a promising technique with the potential to accelerate genetic improvement by hastening the dissemination of superior genetic resources in a population. The pig is not only a biomedical model and xenotransplant donor due to its physiological similarities to humans, but also an economically important livestock species [1,2]. Although successful pig cloning was first reported in 2000 [3,4], the cloning technique has not yet been widely used in the animal breeding field because of its low efficiency, poor public acceptance of products, and limited assessment of cloned animals’ performance. 

To date, the majority of cloning researches have focused on the improvement of cloning efficiency by optimizing cloning protocols [5,6,7] and regulating epigenetic reprogramming during SCNT [8,9,10]. In addition, several studies have demonstrated that the products derived from clones and their progeny are as safe as those derived from conventionally bred pigs and can enter food supply markets [11,12,13]. In the pig breeding industry, the selection of superior boars for breeding would accelerate genetics improvement in population and produce elite progeny to bring great economic benefit. However, the limited semen production of selected superior boars cannot totally satisfy the intensive breeding program of a large population. One of the strategies to solve this problem is to use semen from boars with relatively inferior performance, but this strategy would notably reduce the benefits of selection and production. How to maximize the dissemination of the genetic resources of superior boars by artificial insemination (AI) is of great interest to livestock breeders. The most promising strategy is multiplying superior boar by cloning. Although clones present a higher rate of malformations or death among fetal and newborn piglets than non-clones [14,15,16], the abnormal phenotypes of surviving clones do not appear to be transmitted to the next generation [17,18]. Therefore, surviving clones, even those with minor malformations, still have the potential to be used as breeding pigs. However, the further assessment of clones, especially the comparison with conventionally bred pigs, needs to be performed. It is necessary to investigate whether surviving clones can grow under normal management conditions, produce semen with normal quality and subsequently exhibit reproductive capability. More importantly, clones of superior boars are expected to conserve the genetic merits and produce progeny with greater growth performance. Thus, the growth performance of the progeny of clones is an important indicator of cloning in practical application. Currently, several studies have compared the growth and reproductive performances of clones and their progeny with those of conventionally bred pigs [19,20,21,22]. Considering that the limited population size of clones and their progeny may lead to false negatives in studies on the difference between clones and conventionally bred pigs, the sample size must be increased to draw a reliable conclusion. Pietrain pigs are widely used as boars to produce lean-type swine because of their high growth rate and lean fat rate, but information on the performance assessment of clones and their progeny is still lacking.

Our previous studies demonstrated that the hematological and biochemical characteristics are not different from those of non-clones [12]. In the present study, we extended to compare the growth performance, semen quality and reproductive performance of clones with those of conventionally bred boars for breeding. Furthermore, the clones and non-clones were randomly bred with the Duroc sows to produce the F1 progeny for commercial production and their growth performance was tested. Our results could provide insights into the long-term effects of cloning and evidence for the further application of clones in commercial pig production.

## 2. Materials and Methods

### 2.1. Ethics Statement

This study was processed in accordance with the Animal Care Committee of South China Agricultural University, Guangzhou, China (approval number #SYXK2014-0136) [23]. All pigs were produced and euthanized by following the Regulations for the Administration of Affairs Concerning Experimental Animals (Ministry of Science and Technology, China, revised June 2004) [12]. 

### 2.2. Animals

This study was constituted by three groups of comparisons. The donor group, which is constituted by donor boars (superior boar) and boars ranked lower in the population (control boar). Clone group, which is constituted by clones and non-clones (progeny of control boar). Progeny group, which is constituted by the progeny of clones and the progeny of non-clones. Donor boars are the providers of ear fibroblast cells for cloning. Control boars are age- and breed-match with donor boars and ranked lower in the breeding pig population. Non-clones are age-match with clones and the progeny of control boar produced by AI. The progeny of non-clones is age-match with the progeny of clones and produced by conventional AI. All pigs in a comparative group were fed standard diet three times each day and watered ad libitum under standard and housed in the same facility. 

### 2.3. Donor Cell Isolation, Ovary Collection, and Oocyte Maturation

Fibroblast cells were isolated from the ears of four superior purebred Pietrain boars as previously described [24]. In a humidified atmosphere of 5% CO_2_ and 95% air, we cultured fibroblasts cells in DMEM (Gibco BRL, Grand Island, NY, USA) supplemented with 10% (*v/v*) FBS (Gibco BRL, Grand Island, NY, USA) at 39 °C for 8–10 days [25]. Cultured cells at passages 3–8 were used for SCNT. We collected recipient oocytes from hybrid gilts (Duroc × Yorkshire × Landrace) in a local slaughterhouse. Cumulus oocyte complexes (COCs) were collected from antral follicles and washed with tissue culture medium 199 (Gibco BRL, Grand Island, NY, USA) supplemented with 3.05 mM d-glucose, 0.91 mM sodium pyruvate, 0.1% (*w/v*) polyvinyl alcohol, 0.57 nM cysteine, 0.5 µg/mL LH, 0.5 µg/mL FSH, 10% (*v/v*) porcine follicular fluid, 75 µg/mL penicillin G, and 50 µg/mL streptomycin, 10 ng/mL epidermal growth factor [24]. After incubated for 1.5 days, the oocytes of COCs were released, and matured oocytes with the first polar body (PB) were used for cloning [25].

### 2.4. SCNT

The SCNT experiment was performed in accordance with our previous study [24]. In brief, we used a micropipette to hold the zona pellucida of the oocyte without cumulus. The first PB and adjacent cytoplasm with most chromosomes were squeezed out with a needle that was used to make a slit near the first PB. A fibroblast cell was inserted into the perivitelline space of enucleated oocytes with a smooth surface through the same slit [24]. After culture in PZM3 medium [26] at 39 °C for 1.5 h, the fusion of oocyte–donor cell complexes was performed via two successive DC pulses at 1.2 kV/cm for 30 µs [27]. 

### 2.5. Embryo Transfer 

Cloned embryos were examined to remove dead embryos and abnormally cleaved embryos after cultured at 39 °C for 20 h in PZM3 medium. A total of 250 purebred Large White sows were used as embryo recipients. Recipient sows with natural standing estrus were inducted to anesthetize with ketamine (25 mg per kg of body weight) and xylazine (1.1 mg per kg of body weight) [25]. The anesthetized status was maintained by 3% isoflurane [25]. After recipients’ oviduct was exposed via surgery, we placed the cloned embryos with 0.1 mL of culture medium into the oviduct of the recipients by using a 1 mL syringe [25]. 

### 2.6. Analysis of Recipient Pregnancy and Delivery of Cloned Piglets

Approximately 1 month after the reconstructed embryo had been transferred into the recipient sow, we used an ultrasound machine to monitor the pregnancy status of all recipients. The recipients were treated with a prostaglandin analogue (200 μg/recipient) if they did not deliver past gestation day 114 [25]. Approximately 24 h after the injection, a caesarean section was performed to deliver if recipient sows were still hard to farrow. Then, the number of newborn cloned piglets and dead fetuses in each litter was recorded. Pregnancy rate is equal to the number of pregnant recipient sows divided by the number of all recipient sows; farrowing rate is equal to the number of farrowed recipient sows divided by the number of all recipient sows; average litter size is equal to the number of all born cloned piglets divided by the number of farrowed recipient sows; developmental rate of cloned embryos is equal to the number of born cloned piglets divided by the number of cloned embryos of all recipient sows [25].

### 2.7. Measurement of Growth Performance 

Body length was measured as the distance from the midpoint of the forehead to the root of the tail along the topline. Body height was measured as the vertical distance from the top of the shoulder to the ground. Average weight gain was measured ranging from ~30 kg to ~100 kg. Backfat thickness was measured at the 10th and 11th rib interface along the midline by using a sliding caliper. Bodyweight and backfat thickness were adjusted by following the protocol of a previous study [28]. The relative coefficient of variation (C.V.) in the growth performance profiles for clones was calculated by the C.V. of clones divided by non-clones. Relative values of ≤ 1 indicate reduced variability for clones.

### 2.8. Evaluation of Semen Quality

The training of boars was started when they were 8 months old. We respectively collected 507 semen and 563 semen from 19 clones and 28 non-clones (clone group) by using the gloved-hand technique and transported samples to the laboratory at 37 °C within 30 min. Semen volume was measured by using a precision electronic balance (assuming 1 mL of semen equaled 1 g of semen). After filtration, the collected fresh semen samples were preliminarily examined under a microscope, and the semen extender PRIMEXcell (IMV Technologies, L’Aigle, France) was used to dilute semen by two- to four-fold. The parameters of semen quality, including sperm motility, abnormal sperm percentage, and sperm concentration, were detected via a hemocytometer counting under a 100-fold phase-contrast microscope as previously described [29]. Abnormal sperm exhibited head or tail defects, such as a misshapen head or double tail.

### 2.9. Measurement of Reproductive Performance

The semen from 21 clones was artificially inseminated into 1380 conventionally bred Duroc sows to produce the progeny of clones. The semen from 25 non-clones was artificially inseminated into 1437 conventionally bred Duroc sows to produce the progeny of non-clones. All sows were age-match from the same population and housed in the same environment. After Pietrain boars became sexually mature, semen samples were collected and diluted to a final concentration of 3 billion spermatozoa in 80 mL of the semen extender PRIMEXcell (IMV Technologies, France). Purebred Duroc sows in natural estrus were inseminated two times with an interval of 8–12 h between each insemination [25]. Recipient pregnancy was diagnosed, and cloned piglets were delivered as described above.

### 2.10. Progeny Test

We examined the growth performance of 466 progeny of the clones and 822 progeny of the non-clones (progeny group) to investigate whether the elite genetic resources of clones can be transmitted to their progeny. In this study, the type score was the total score of head shape, hoof, cannon circumference and limb. Predicted percent lean was calculated in accordance with a previous study [30]. Bodyweight was recorded from birth to ~60 kg body weight and then used to calculate average daily gain. Backfat thickness was measured at the 10th and 11th rib interface along the midline by using a sliding caliper. The relative coefficient of variation (C.V.) in the growth performance profiles for the progeny of clones was calculated by the C.V. of the progeny of clones divided by the progeny of non-clones. Relative values of ≤ 1 indicate reduced variability for the progeny of clones. 

### 2.11. Statistical Analysis

The normality distribution of all traits was evaluated using Shapiro–Wilk’s test. Independent Student’s *t*-test and one-way analysis of variance (ANOVA) were respectively employed for the parameters with two groups and more than two groups that following the normal distribution. Non-parametric analogous Mann–Whitney U test and Kruskal–Wallis test were respectively employed for the parameters with two groups and more than two groups that not following the normal distribution. Percentage data were arcsine-transformed before analysis. For the analyses of semen quality and reproductive performance, we used boar as the experimental unit with ejaculates as repeated measure. All data analysis in this study were performed by using statistical package IBM SPSS Statistics software [31]. All data shown in the tables and figures are the mean ± standard error of the mean.

## 3. Results

### 3.1. Donor Boar Selection

**Figure 1:**
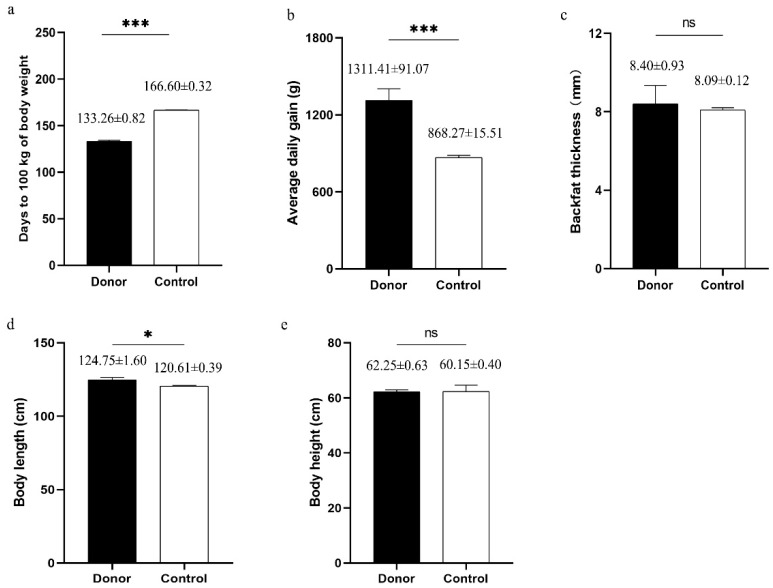
Growth performance of donor boars (*n* = 4) and control boars (*n* = 36). (**a**) Adjusted days to 100 kg of body weight. (**b**) Average daily gain between adjusted 30 kg to 100 kg of body weight; (**c**) Backfat thickness adjusted to 100 kg of body weight; (**d**) Body length. (**e**) Body height. * stands for *p* ≤ 0.05, *** stands for *p* ≤ 0.001. ns stands for nonsignificant differences.

Boar selection is focused on the growth rate in practical application. In this study, four Pietrain boars were selected on the basis of the comparison of the growth performance in breeding pig population to provide donor cells for cloning. As shown in Figure 1, donor boars exhibited significantly greater average daily gain and body length, and fewer days to 100 kg of body weight than those of the other boars in the population (control boar). This result indicated that selected boars were superior and valuable to cloning.

### 3.2. Production of Clones

A total of 59,061 SCNT embryos cloned from four superior Pietrain boars were transferred to 250 sows in spontaneous estrus. As shown in Table 1, our data showed that the developmental rate of cloned embryos (cloning efficiency) was 0.67%, and no significant difference was observed among the four groups. The average pregnancy rate was 74.40%, and the farrowing rate was 44.80%, indicating that a high pregnancy loss appeared in the cloned embryos. The average litter size was 3.28, and it significantly differed among the compared groups. The clones exhibited a high rate of malformations or death. The entire lists of record, including different types of malformations in piglets, are presented in Supplementary Table S1. 

### 3.3. Growth Performance 

**Figure 2:**
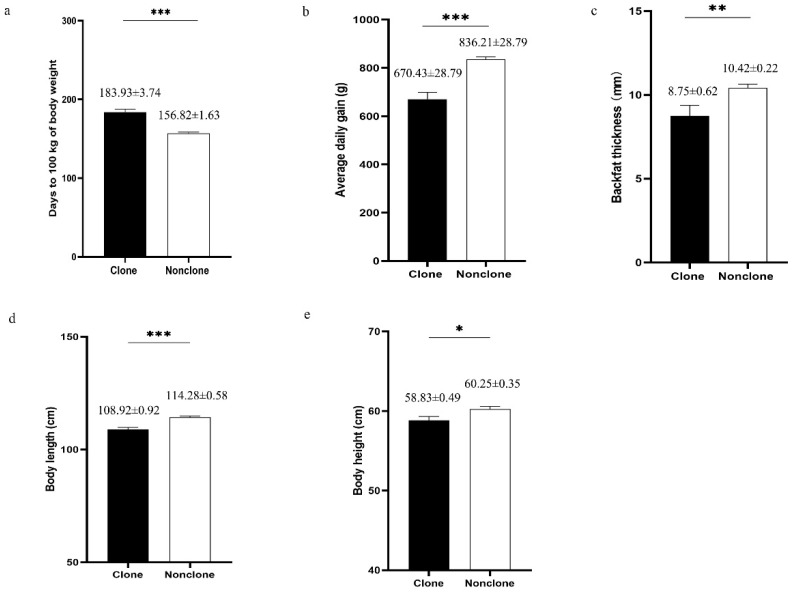
Growth performance of clones (*n* = 12) and non-clones (*n* = 36). (**a**) Adjusted days to 100 kg of body weight. (**b**) Average daily gain between adjusted 30 kg to 100 kg of body weight; (**c**) Backfat thickness adjusted to 100 kg of body weight; (**d**) Body length. (**e**) Body height. * stands for *p* ≤ 0.05, ** stands for *p* ≤ 0.01, *** stands for *p* ≤ 0.001. ns stands for nonsignificant differences.

Five growth performance parameters of 12 clones and 36 non-clones (clone group) were investigated to compare their growth statuses. As shown in Figure 2, the parameters, including average daily gain, body weight, body length, body height, and backfat thickness of the clones were significantly lower than those of the non-clones. This result indicated that cloned Pietrain boars grew slower and exhibited a smaller body size than the non-clones. The detailed records can be found in Supplementary Table S2.

### 3.4. Semen Quality 

A total of 507 and 563 semen samples were collected from 19 clones and 28 non-clones (clone group), respectively, to evaluate semen quality. As shown in Table 2, no significant difference in semen volume, sperm motility, sperm concentration and percentage of abnormal sperm was observed between the two groups. The entire records are displayed in Supplementary Table S3.

### 3.5. Breeding Test

A total of 21 clones and 25 non-clones (clone group) were randomly bred with Duroc sows to examine the effect of cloning on reproductive performance. We had respectively collected 1380 litters of the clones and 1437 litters of the non-clones. As shown in Table 3, clones had more still-born piglets than non-clones (*p* ≤ 0.05), but the number of total piglets (including the dead fetuses), born-alive piglets, weaned piglets, born-weak piglets, mummified fetuses and the litter weight in each litter of the clones did not differ from those in each litter of the non-clones. In conclusion, we found that compared with non-clones, clones showed similar reproductive performance in terms of major parameters, except for more still-born piglets per litter. The detailed results of the breeding test are listed in Supplementary Table S4.

### 3.6. Progeny Test

**Figure 3:**
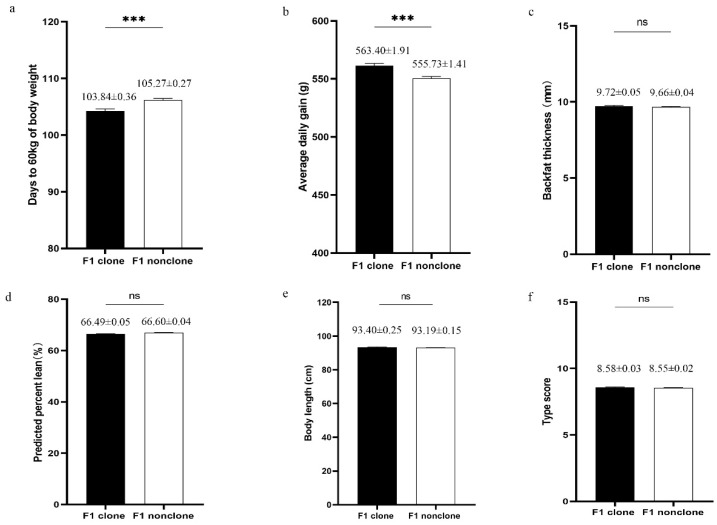
Growth performance of F1 progeny of clones (*n* = 466) and F1 progeny of non-clones (*n* = 822). (**a**) Adjusted days to 60 kg of body weight; (**b**) Average daily gain from birth to adjusted 60 kg of body weight; (**c**) Backfat thickness adjusted to 60 kg of body weight; (**d**) Predicted percent lean. (**e**) Body length; (**f**) Type score. *** stands for *p* ≤ 0.001, ns stands for nonsignificant differences. F1 clone stands for the F1 progeny of clones. F1 nonclones stands for the F1 progeny of non-clones.

The growth performance was compared between 466 progeny of the clones and 822 progeny of the non-clones (progeny group). As shown in the Figure 3, backfat thickness, lean percentage, body length, and type score were similar between the progeny of the clones and the non-clones. However, the body weight and average daily gain of the progeny of the clones were significantly higher than those of the non-clones (*p* ≤ 0.001). Thus, we found that the progeny of the clones exhibited greater growth performance than those of the non-clones. The detailed results of the progeny test are shown in Supplementary Table S5.

### 3.7. Coefficient of Variation Analysis

**Figure 4:**
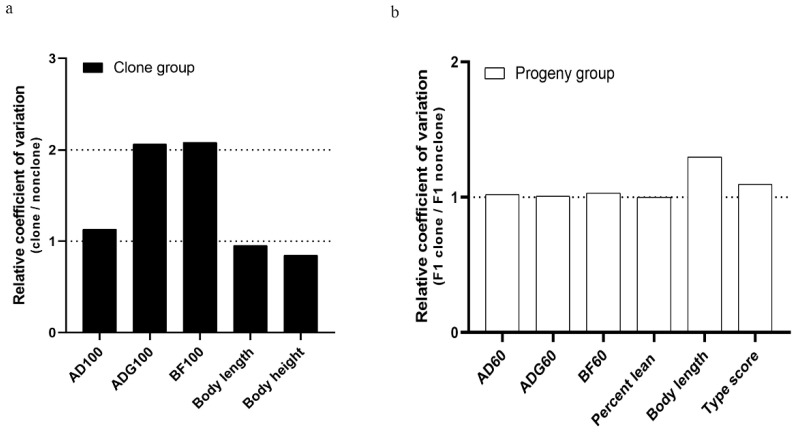
Variability of growth performance parameters in the clone group and progeny group. (**a**) Relative coefficient of variation in clones and non-clones (clone group). (**b**) Relative coefficient of variation in the F1 progeny of clones and the F1 progeny of non-clones (progeny group). AD100, adjust days to 100 kg body weight. ADG100, average daily gain between adjusted 30 kg to 100 kg of body weight. BF100, backfat thickness adjusted to 100 kg of body weight. AD60, adjust days to 60 kg body weight. ADG60, average daily gain from birth to 60 kg of body weight. BF60, backfat thickness adjusted to 60 kg of body weight. F1 clone stands for the F1 progeny of clones. F1 nonclones stands for the F1 progeny of non-clones.

In this study, the relative coefficient value of growth performance parameters of clones and their progeny was calculated to investigate whether cloning manipulation reduced variations between individuals. As shown in Figure 4, although reduced variation in clones was observed in several parameters, the body weight, average daily gain and backfat thickness in clones appeared greater variations than non-clones. In the F1 generation, however, the variations of most parameters in the progeny of clones are similar to the progeny of non-clones except the greater variations in body length and type score. These results showed that although cloning increased the variability of some traits associated with growth performance, there was a decreased tendency in the next generation.

## 4. Discussion

The artificial insemination (AI) technique is widely used for the reproduction of more than 90% of sows in most pork-producing countries [32]. Using fewer genetically high-ranking boars to provide semen for reproduction by AI can accelerate the expansion of elite genetic resources in the population and result in a better profitability of each boar ejaculate [33]. Thus, how to make the limited semen production of selected boars to be enough for the large population of sows has attracted increasing attention. The cloning of superior boars has the potential to conserve the genetic merits of superior boars and could be used to provide more semen for reproduction. However, the performance of cloned boars remains for further investigation. In this study, we selected four superior Pietrain boars for their excellent growth performance in population and used them to produce clones via SCNT. Our study made a series of comparisons between the clones and age-matched non-clones. Such a comparison was also performed in previous studies to assess the applicability of cloned animals [12,21,34,35,36,37,38]. We aim to investigate whether clones and their progeny keep behaving better than the “normal boars”, which is important for the application of cloning to expand the elite genetic resources and improve the economic value of selection and production in the pig industry.

In this study, we found that the cloning efficiency was very low but similar to the previous studies (1–3%) [39,40]. Cloned Pietrain boars grew slower and exhibited smaller body size than the conventionally bred boars, suggesting that cloning manipulation might impair the growth performance of animals. Similarly, cloned cows have been found to exhibit a lower growth rate [35] and even reach puberty later than non-cloned cows [36]. However, these results were inconsistent with other previous studies, indicating that clones grow normally and have similar characteristics to those of non-clones [19,20,21]. In addition, Kawarasaki et al. demonstrated that the body weights of 3- to 7-month-old clones are slightly higher than those of conventionally bred controls [34]. We inferred that differences in the growth performance of the clones might be caused by differences in pig breeds and variabilities resulting from random epigenetic dysregulation during cloning [41]. Cloning syndrome appears quite common in cloned animals, especially in cattle, whose typical symptoms were a higher rate of pregnancy loss, higher birth weights, higher rate of post-natal mortality and higher rate of malformations [14,42]. This syndrome is a continuum to affect individual performance throughout the neo-natal period and adulthood in cattle [43]. In our study, the clones also exhibited high rate of postnatal malformation, including macroglossia, incomplete digestive system, limb hyperflexion and hypoplastic testis. These defects may be caused by the abnormalities of epigenetic status, such as DNA methylation [44,45,46], gene imprinting [47,48,49], and histone modifications [50,51]. However, the majority of malformed piglets in our study died in the neonatal period due to the malformations in the musculoskeletal system, and approximately half of the newborn piglets could survive, which were consistent with the previous study [14]. These results could support the application for the cloning of superior pigs although further efforts are needed to reduce malformations in cloned piglets.

Semen quality is affected by a number of factors, such as the age of collection [34], season [52], breed [53], and individual response to the environment [54]. Current studies on the semen quality of clones and non-clones indicate that cloning technology can also affect semen quality [21,34], but contradictory results are observed in some parameters. Chen et al. reported that there is no significant difference in semen volume and sperm concentration [21], whereas Kawarasaki et al. demonstrated that clones have higher semen volume and sperm concentration than non-clones [34]. In this study, the evaluation of semen quality on more than 1000 semen samples from the clones and non-clones showed no significant difference. The semen motility was more than 70% and the percentage of abnormal sperm was less than 10%, which satisfied the standard for reproduction [32]. To further investigate the feasibility of using semen from clones in production, we randomly inseminated the semen from the clones and the non-clones into Duroc sows to produce crossbred progeny for commercial production. We found that the clones had a similar reproductive performance to the non-clones, which was in agreement with previous reports on pigs [19,22,34] and cattle [55,56]. Furthermore, considering that the cells nuclear for cloning were derived from superior boars, clones were expected to conserve the elite genetic resources and thus produce progeny with greater growth performance. Our data showed that the progeny of the clones presented a greater growth rate than that of the non-clones, although cloning caused defects in the growth performance of the clones. The transiently poor growth performance of the clones was not transmitted to their progeny. This “correction” in the next generation has also been reported in cloned mice [17,18] and cattle [57,58], suggesting that inappropriate epigenetic modification can be reprogrammed during the formation of germlines. In addition, our study demonstrated that cloning increased variability associated with some traits of growth performance, but these high-variation traits also appeared as a “correction” in the F1 progeny of the clones, as reported in the blood parameters of cloned pigs [41,59]. Taken together, we support that the cloning of superior boars could be used to increase economic benefits by producing commercial pigs with superior performance.

## 5. Conclusions

Our results demonstrated that although cloning manipulation caused some defects in growth performance, clones could produce semen with normal quality and possess normal reproductive performance. The F1 progeny of the clones exhibited greater growth performance than that of the non-clones because of the elite genetic resources passed by superior donor boars. We support that the cloning of superior boars can be applied to expand the elite genetic resources and improve the economic value of selection and production in the pig industry. 

## Figures and Tables

**Table 1:** Summary of the production of cloned Pietrain boars.

Parameters	Group				Average
	Boar 1	Boar 2	Boar 3	Boar 4	
No. of recipient sows	50	148	27	25	62.50
No. of cloned embryo	11,068	36,483	5771	5739	14,765
Pregnancy rate (%)	76.00	72.97	77.78	76.00	74.40
Farrowing rate (%)	48.00	45.95	33.33	44.00	44.80
No. of total piglets	91	197	31	48	92
No. of born-alive piglets	59	155	23	38	69
No. of weaned piglets	33	56	8	14	28
Average litter size	3.79 ± 0.37 ^b^	2.89 ± 0.18 ^a^	3.44 ± 0.50 ^a,b^	4.36 ± 0.80 ^b^	3.28 ± 0.17
Developmental rate of cloned embryos (%)	0.86 ^a^	0.58 ^a^	0.56 ^a^	0.98 ^a^	0.67

Values in the same row labeled without a common superscript letter (a and b) stand for significant differences among groups (*p* ≤ 0.05). Values in the same row labeled with the common superscript letter stands for no significant differences among groups (*p* ≥ 0.05).

**Table 2:** Semen quality of cloned and non-cloned Pietrain boars.

Parameters	Clone (*n* = 19)	Nonclone (*n* = 28)	*p* Values
No. of ejaculates	507	563	
Semen volume (mL)	232.95 ± 12.17	237.47 ± 9.91	0.774
Sperm motility	0.71 ± 0.02	0.71 ± 0.02	0.128
Sperm concentration (10^8^/mL)	2.77 ± 0.18	2.58 ± 0.47	0.544
Percentage of abnormal sperm (%)	8.42 ± 0.48	8.48 ± 0.58	0.950

**Table 3:** Reproductive performance of cloned and non-cloned Pietrain boars.

Parameters	Clone (*n* = 21)	Nonclone (*n* = 25)	*p* Values
No. of sows	1380	1437	
Total piglets/litter	9.90 ± 0.07	10.19 ± 0.16	0.117
Born-alive piglets/litter	8.80 ± 0.08	9.14 ± 0.17	0.080
Weak piglets/litter	0.46 ± 0.03	0.41 ± 0.04	0.741
Malformed piglets/litter	0.47 ± 0.05	0.38 ± 0.03	0.072
Mummified piglets/litter	0.51 ± 0.05	0.54 ± 0.05	0.843
Still-born piglets/litter	0.59 ± 0.05	0.51 ± 0.08	0.035
Weaned piglets/litter	7.83 ± 0.06	7.93 ± 0.10	0.225
Litter weight of piglets (kg)/litter	14.27 ± 0.15	14.72 ± 0.20	0.158

## Supplementary Materials

The following are available online at https://www.mdpi.com/2076-2615/10/11/2053/s1. All supplementary data generated during this study are included in supplementary information files. Table S1: Summary of production of cloned Pietrain boars; Table S2: Growth performance of cloned and non-cloned Pietrain boars; Table S3: Semen quality of cloned and non-cloned Pietrain boars; Table S4: Reproductive performance of cloned and non-cloned Pietrain boars; Table S5: Growth performance of the progeny of cloned and non-cloned Pietrain boar.
